# Succinic Semialdehyde Dehydrogenase Deficiency: In Vitro and In Silico Characterization of a Novel Pathogenic Missense Variant and Analysis of the Mutational Spectrum of *ALDH5A1*

**DOI:** 10.3390/ijms21228578

**Published:** 2020-11-13

**Authors:** Heiko Brennenstuhl, Miroslava Didiasova, Birgit Assmann, Mariarita Bertoldi, Gianluca Molla, Sabine Jung-Klawitter, Oya Kuseyri Hübschmann, Julian Schröter, Thomas Opladen, Ritva Tikkanen

**Affiliations:** 1 Department of General Pediatrics, Division of Neuropediatrics and Metabolic Medicine, University Children’s Hospital Heidelberg, 69120 Heidelberg, Germany; Heiko.Brennenstuhl@med.uni-heidelberg.de (H.B.); Birgit.Assmann@med.uni-heidelberg.de (B.A.); Sabine.Jung-Klawitter@med.uni-heidelberg.de (S.J.-K.); Oya.KuseyriHuebschmann@med.uni-heidelberg.de (O.K.H.); Julian.Schroeter@med.uni-heidelberg.de (J.S.); 2 Institute of Biochemistry, Medical Faculty, University of Giessen, 35392 Giessen, Germany; Miroslava.Didiasova@biochemie.med.uni-giessen.de; 3 Department of Neuroscience, Biomedicine and Movement, Section of Biological Chemistry, University of Verona, Strada Le Grazie, 8, 37134 Verona, Italy; Mita.Bertoldi@univr.it; 4 Department of Biotechnology and Life Sciences, University of Insubria, via J.H. Dunant 3, 21100 Varese, Italy; Gianluca.Molla@uninsubria.it

**Keywords:** inherited metabolic disease, succinic semialdehyde dehydrogenase deficiency, γ-amino butyric acid, γ-hydroxybutyrate, mutational spectrum

## Abstract

Succinic semialdehyde dehydrogenase deficiency (SSADHD) is a rare, monogenic disorder affecting the degradation of the main inhibitory neurotransmitter γ-amino butyric acid (GABA). Pathogenic variants in the *ALDH5A1* gene that cause an enzymatic dysfunction of succinic semialdehyde dehydrogenase (SSADH) lead to an accumulation of potentially toxic metabolites, including γ-hydroxybutyrate (GHB). Here, we present a patient with a severe phenotype of SSADHD caused by a novel genetic variant c.728T > C that leads to an exchange of leucine to proline at residue 243, located within the highly conserved nicotinamide adenine dinucleotide (NAD)^+^ binding domain of SSADH. Proline harbors a pyrrolidine within its side chain known for its conformational rigidity and disruption of protein secondary structures. We investigate the effect of this novel variant in vivo, in vitro, and in silico. We furthermore examine the mutational spectrum of all previously described disease-causing variants and computationally assess all biologically possible missense variants of *ALDH5A1* to identify mutational hotspots.

## 1. Introduction

Succinic semialdehyde dehydrogenase deficiency (SSADHD) is a rare monogenic disorder (OMIM: #271980) of the degradation pathway of the key inhibitory neurotransmitter of the central nervous system, γ-amino butyric acid (GABA). Variants of the *ALDH5A1* gene (6p22.3, GRCh38.p13: 24,494,969–24,537,207) are known to cause a loss of function of the mitochondrial succinic semialdehyde dehydrogenase (SSADH) and were identified in 1981 to be the underlying cause of SSADHD [1]. Affected individuals become symptomatic within the first years of life, showing developmental delay (98.5% of all cases), intellectual disability (95.6% of all cases), muscular hypotonia (83.3% of all cases), behavioral difficulties (69.1% of all cases), and variable epileptic semiologies (50% of all cases), some of which are difficult to treat. Individual reports of sudden unexpected death in epilepsy (SUDEP) in SSADH patients exist and indicate an incidence of 15% [2,3,4,5,6]. The diagnosis is based on the biochemical analysis of γ-hydroxybutyrate (GHB) concentrations in the urine, although it can also be measured in the blood and the cerebrospinal fluid (CSF). Measurement of SSADH enzymatic activity in lymphocytes or sequencing of the *ALDH5A1* gene is used to confirm the diagnosis [7]. The therapeutic approach is symptomatic, mainly focusing on behavioral problems and epilepsy, with no causative therapy available to date [8].

**Figure 1:**
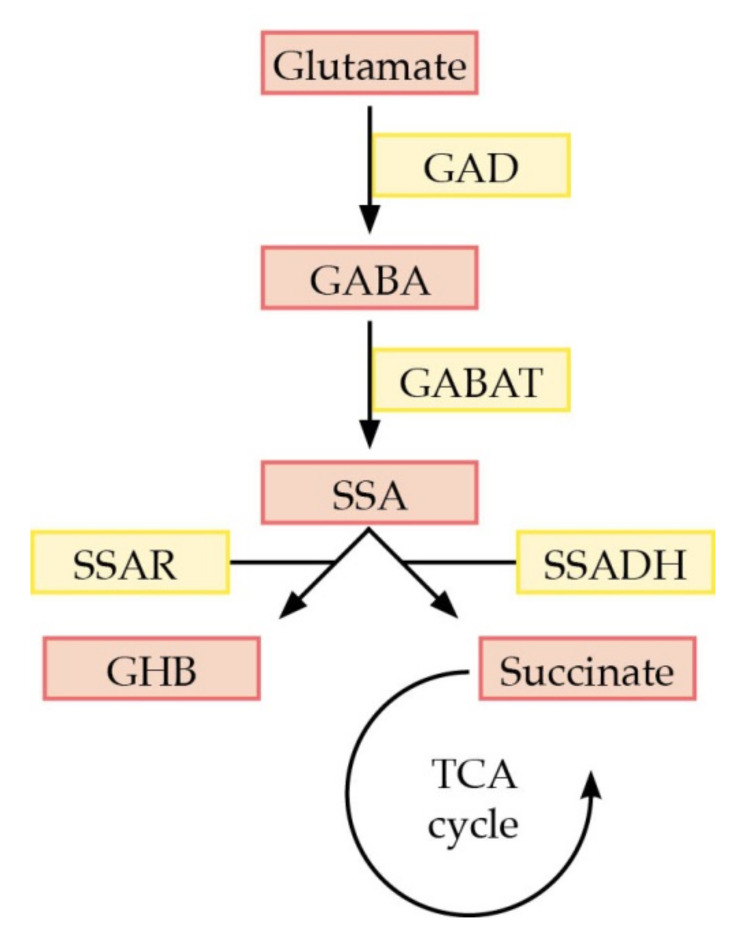
Metabolic pathway of γ-amino butyric acid (GABA) degradation. Abbreviations: GAD = glutamate decarboxylase, GABAT = GABA transaminase, SSA = succinic semialdehyde, SSADH = succinic semialdehyde dehydrogenase, SSAR = succinic semialdehyde reductase, GHB = γ-hydroxybutyrate, TCA = tricarboxylic acid cycle.

SSADH (EC 1.2.1.24) is a tetrameric mitochondrial nicotinamide adenine dinucleotide (NAD^+^)-dependent oxidoreductase that converts succinic semialdehyde (SSA) to succinic acid for its further utilization in the tricarboxylic acid (TCA) cycle (Figure 1). Due to the enzymatic dysfunction in SSADHD, SSA and GABA accumulate in tissues, CSF, blood, and urine, leading to a subsequent conversion of SSA to GHB by succinic semialdehyde reductase (SSR) [9]. The *ALDH5A1* gene is located on chromosome 6, comprises 10 exons, and produces an open reading frame of 535 amino acids. The first 47 amino acids represent a mitochondrial targeting signal, followed by an NAD^+^-cofactor-binding domain (residues 48–173, 196–307, and 509–524), an oligomerization domain (residues 174–195, 525–535), and a catalytic domain (residues 308–508). Thus, the percentage distribution of the individual domains within the protein is as follows: 48% NAD^+^ binding domain, 38% catalytic domain, 9% mitochondrial targeting signal and about 5% oligomerization domain. The catalytic domain contains a catalytic loop with a reversible disulfide bond between residues Cys340 and Cys342, forming a dynamic redox switched loop that inhibits the entrance of a substrate in the oxidized form of the enzyme [10,11].

## 2. Results

### 2.1. Clinical Synopsis

The female patient described herein was born at term to non-consanguineous parents after an uneventful pregnancy. The patient’s birth weight was 2540 g (2nd percentile), length 49 cm (14th percentile), and head circumference 35 cm (59th percentile). After an inconspicuous newborn period, developmental delay was noted at 4 months of age due to the absence of grasping. At the age of 8 months, the patient was presented to a neuropediatrician for further evaluation, revealing hypotonia and a mild developmental delay. Semi-quantitative analysis of organic acids in the urine revealed increased levels of GHB and γ-butyrolacton. Levels of GABA and GHB in the CSF were also elevated, whereas SSADH enzymatic activity in lymphocytes was absent. Magnetic resonance imaging of the brain was done at the age of 1 year and revealed occipital white matter changes with delayed myelination. Molecular genetic investigation was not done at this time.

During infancy, the main clinical findings were truncal hypotonia and diurnal changes of the vigilance. Milestones of gross motor development were achieved with delay, with free sitting at one year and independent walking at the age of 4. The patient’s speech comprehension was significantly reduced, and active speaking since the age of six years has been limited to five-word sentences. At the age of six years, the patient developed a wide-based gait without further cerebellar signs, which was caused by the combination of hypotonia and some interfering stiffening of the legs—probably due to a mild generalized dystonia or a more unspecific “central tone-coordination disorder”. Focal and secondary bilateral seizures with and without loss of consciousness started at the age of six years. The electroencephalogram revealed multifocal spike-wave complexes and other epileptic discharges. Over the years, most seizures occurred during sleep or during the awaking period. The epilepsy was therapy-resistant with the assumed side-effects to lamotrigine, oxcarbamazepine, levetiracetam (also in combination), and a short valproate trial. The implantation of a vagus nerve stimulator was followed by weeks without seizures. However, upon a following escalation, acetazolamide was started and attempted to be replaced by topiramate. Weaning trials of acetazolamide were followed by enhanced seizure activity.

To date, under topiramate and acetazolamide, the patient still suffers from recurring focal seizures with eyelid flutter, trembling of both hands, sometimes with subsequent generalization, and there is a regular need for emergency medication to avoid generalized seizures and status epilepticus. Moreover, the patient is being treated with melatonin due to a sleep disorder that can be classified as parasomnia with repeated nocturnal awakening and confusional arousals. The patient, now aged 23 years, suffers from an autism spectrum disorder with repetitive movements, anxiety, and social withdrawal, characteristic for SSADH deficient patients. Her cognitive abilities are heterogeneous and impaired by autistic and possibly some features of dyspraxia.

The family history of the patient’s mother was unremarkable; whereas the patient’s father suffers from recurrent seizures that have not been further evaluated. The paternal uncle of the patient suffers from an autism spectrum disorder that has not been further investigated. The patient has one healthy brother.

### 2.2. Biochemical Data and Sequence Analysis

**Figure 2:**
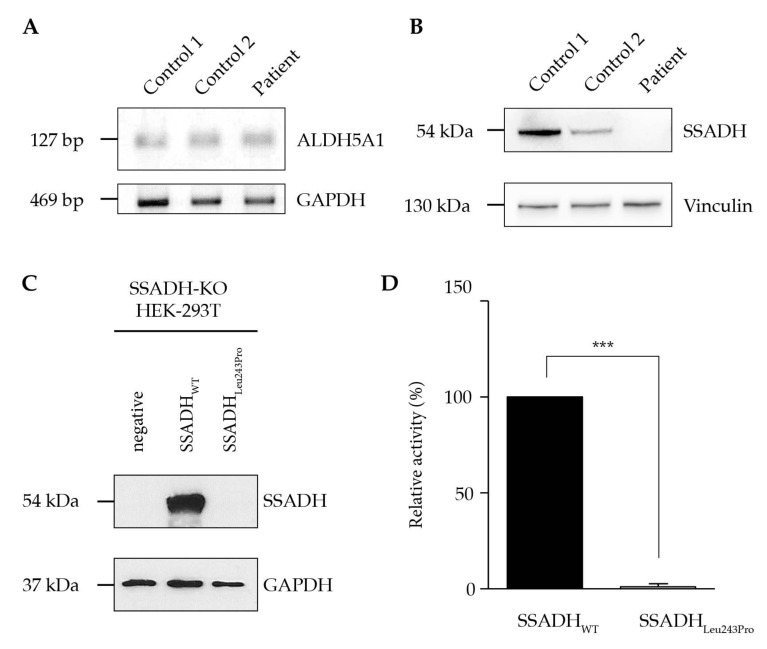
Biochemical characterization of the Leu243Pro variant. (**A**) Semi-quantitative polymerase chain reaction (PCR) analysis of *ALDH5A1* gene expression of two independent control subjects and the patient harboring the c.278G > T and c.728T > C variants. (**B**) Western blot analysis of succinic semialdehyde dehydrogenase (SSADH) protein expression in dermal fibroblasts. (**C**) SSADH-deficient HEK-293T cells (SSADH-KO-HEK-293T) were transiently transfected with either wildtype (WT) SSADH or p.Leu243Pro constructs. After 48 h, cell lysates were prepared and subjected to Western blotting (**C**) and SSADH enzyme activity assay (**D**). (**C**) Representative Western blot analysis of SSADH protein levels. Glyceraldehyde 3-phosphate dehydrogenase (GAPDH) was used as a loading control. *n* = 3. (**D**) SSADH enzyme activity was assessed fluorometrically. WT activity was set to 100%. Data are expressed as mean ± SD of three independent experiments. Student’s *t* test: *** *p* ≤ 0.01.

A confirmation of the diagnosis was achieved by a complete absence of SSADH enzyme activity in lymphocytes. Analysis of the *ALDH5A1* gene showed heterozygosity for a known missense variant, c.278G > T, leading to a replacement of cysteine to phenylalanine at residue 93 and resulting in a reduced activity of the SSADH enzyme to about 3% when expressed in vitro [12]. A novel variant, c.728T > C, was found, causing an exchange of leucine to proline at residue 243. We used patient-derived dermal fibroblasts to assess the mRNA expression of *ALDH5A1* and found no difference compared to two independent non-SSADHD control subjects (Figure 2A). However, Western blot analysis revealed a nearly complete absence of SSADH protein expression in patient fibroblasts compared to two independent non-SSADHD control samples. Interestingly, the non-SSADHD samples also revealed substantial differences in the expression patterns of the SSADH protein (Figure 2B). An analysis of the sequence homology showed that leucine at position 243 is conserved in all of the 30 mammalian SSADH sequences closest to the human one (data not shown).

To assess the variant’s effect on protein stability, we established an SSADH-deficient HEK293T cell line (SSADH-KO-HEK-293T) using the CRISPR-Cas9 (Clustered Regularly Interspaced Short Palindromic Repeats) technique. Furthermore, using site-directed mutagenesis, we created a p.Leu243Pro plasmid that was used to analyze the protein stability of the mutant protein. Upon transfection of a plasmid carrying the wildtype cDNA into the knockout line, SSADH expression increases, whereas transfection of the mutant construct shows no protein expression. After transfection of the SSADH knockout cell line with the mutant construct, an enzyme activity of only 0.7% of the wildtype could be detected (Figure 2C,D).

### 2.3. In Silico Analysis of Variant Stability

We used the FoldX suite forcefield algorithm to predict the effect of missense variants on protein stability. We computed wildtype protein stability calculations based on the publicly available X-ray crystal structure of reduced SSADH (PDB accession number: 2w8o) [10]. Negative ΔΔ*G_change_* values denote a gain of native-state stability in the variant form, whereas positive ΔΔ*G_change_* values reflect a less stable energy state compared to the wildtype form. The monomers of the p.Cys93Phe and p.Le243Pro variants possess a moderate to high destabilizing ΔΔ*G* of 7.20 and 7.63 kcal/mol, respectively. A total of seven different kinds of tetramers can be obtained by the random combination of these two different single-position variants. The tetrameric SSADH protein consisting exclusively of the p.Cys93Phe missense variant monomers revealed a ΔΔ*G* of 28.09 kcal/mol, meaning it has a destabilizing effect on the wildtype structure, while tetramers of p.Leu243Pro showed even higher values at around 29.36 kcal/mol. Tetramers with all other possible monomer combinations of both variants revealed similar destabilizing properties on SSADH tetramer stability and are shown in Table 1. With regard to the overall high ΔΔ*G* values, it must be assumed that none of the tetrameric variant combinations would lead to an even partially functional SSADH protein.

**Figure 3:**
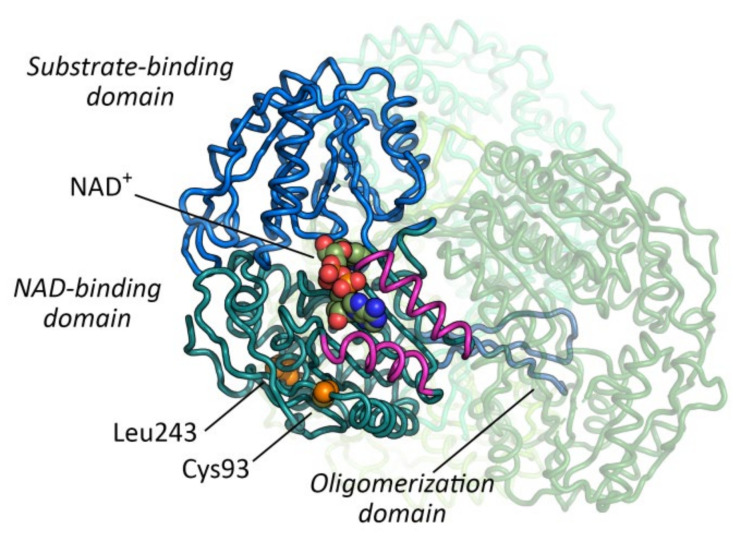
Tetrameric structure of human SSADH (PDB code: 2w8o**).** The substrate-binding domain, nicotinamide adenine dinucleotide (NAD^+^) -binding domain and oligomerization domain of the reference monomer are colored in different shades of blue. The other three monomers are represented by transparent shades of green. The two α-helices in close contact with the adenosine monophosphate moiety of NAD^+^ are colored in purple. Proteins are represented as tubes, while NAD^+^ and the mutated residues (Cys93 and Leu243, αC- αC distance: 20.8 Å) are represented by Van der Waals spheres. The NAD^+^ molecule has been modelled into the NAD^+^-binding domain based on the published structure of the human SSADH in complex with adenosine diphosphate moiety as a ligand (PDB code: 2w8r) [10].

Both missense variants are localized within the structurally and catalytically important NAD^+^ cofactor binding domain, but they are significantly distant from the binding site of the cofactor. The distance of Cys93 and Leu243 from NAD^+^ is ~12 and ~23 Å, respectively. Thus, in agreement with the results from the in silico stability analysis, the effect of the substitutions is protein destabilization rather than a direct effect on the ability of the protein to bind the cofactor. Interestingly, Cys93 is in close contact with an α-helix that, in turn, is important for NAD^+^ binding (Figure 3). Although the Leu243 residue is solvent-exposed, it does not participate to the interface surface between the monomers in the tetramer. Accordingly, an analysis of protein interfaces, surfaces and assemblies (PISA) revealed no difference of either missense variant on oligomerization energy when comparing wildtype and mutant SSADH [13].

### 2.4. SSADH Variant Distribution

**Figure 4:**
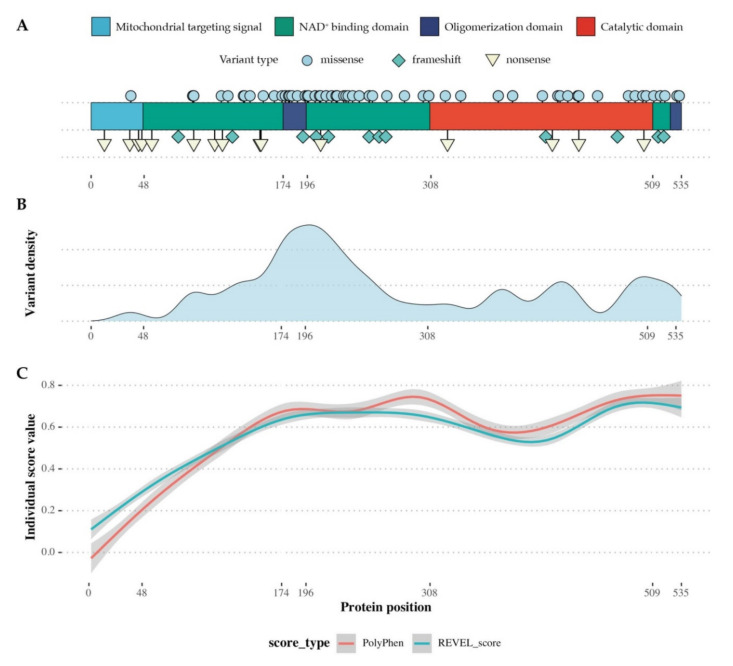
Distribution of disease-causing variants along a linearized model of the SSADH protein. (**A**) Linearized model of the SSADH protein. Functionally important domains are highlighted; symbol-coded variant distribution. (**B**) Assessment of variant density reveals a distinct cluster of pathogenic missense variants in the central region of the SSADH protein and mainly involves the NAD^+^ binding domain and the oligomerization domain. (**C**) Polynomial regression model of two pathogenicity scores (PolyPhen and REVEL) for all possible missense variants along the linearized SSADH protein.

We assessed the distribution of 62 *ALDH5A1* missense variants from 20 publications, three variants from the *Leiden Open Variation Database* (LOVD) [14,15] and the p.Leu243Pro variant first reported here. Missense variants can be found throughout the entire SSADH protein and in all four relevant protein domains. A marked accumulation of missense variants can be observed in the region of amino acids 118–284 within the protein’s NAD^+^ binding domain (53.3%) and in the oligomerization domain (18.3%), while 16 variants have been reported in the catalytic domain (26.7%), and only one variant was found at the *N*-terminal end within the mitochondrial targeting signal (1.7%) (Figure 4).

**Figure A1:**
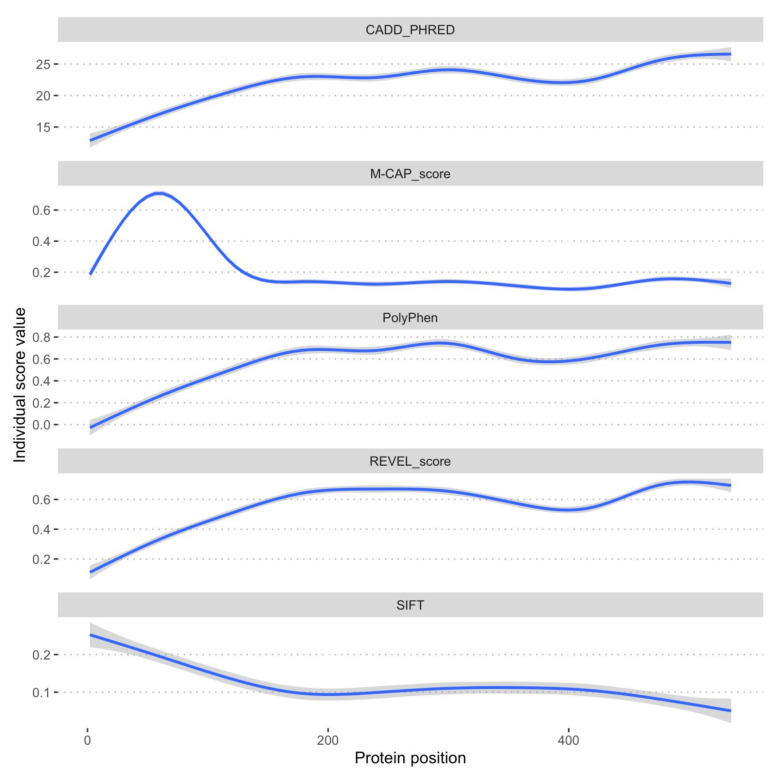
Computational polynomial regression model of 5 pathogenicity scores plotted against the linearized SSADH protein.

We computationally calculated pathogenicity scores for all biologically possible *ALDH5A1* missense variants. For this purpose, we used NM_001080.3 as the source transcript and mutated each base into its three alternate bases. The exchanges that led to a synonymous amino acid sequence were subsequently eliminated. The resulting file was then translated to the gene’s chromosomal sequence and all resulting pathogenicity scores were calculated using Ensemble’s Variant Effect Predictor (VEP) tool. The score values were plotted against the residue position along a linearized SSADH protein using polynomial regression analyses. We found a strong concordance of independent scores with high values, indicating increased pathogenicity along the central and C-terminal part of the linearized SSADH protein, mainly localized to the NAD^+^ binding domain, while scores within the catalytic domain were found to be lower. Overall, similar score tendencies were observed for all established pathogenicity models (Appendix A: Figure A1), underlining the structural and functional importance of the NAD^+^ binding domain and its high susceptibility towards pathogenic variants. Table 2 shows all reported *ALDH5A1* missense variants and their corresponding protein changes known to the literature to date. The NAD^+^ binding domain makes up the largest part of the protein, so it would be expected that most of the pathogenic variants are also found in this domain. In contrast to the missense variants, frameshift and nonsense variants are heterogeneously distributed over the entire length of the protein.

## 3. Discussion

SSADH deficiency describes a rare neurometabolic disease that causes global developmental delay, intellectual disability, hypotonia, and behavioral abnormalities, often classified as an autistic spectrum disorder. A special burden to the patients and families is a sleep disorder with hyperfragmentation of sleep circles (probably as a direct result of the chronic GHB-intoxication), as well as epilepsy. Increased production and urinary excretion of GHB is the key metabolic parameter found in the patients. As illustrated in Figure 1, the brain is chronically exposed to elevated concentrations of GHB and GABA. This is highly relevant to disease pathogenesis, as GHB is also used as a sedative medication and, illegally, as an intoxicant.

Here, we present the case of a 23-year-old female patient with severe developmental abnormalities and behavioral changes typical for SSADHD. Due to the typical constellation of symptoms in early childhood, the diagnosis of SSADH deficiency could be made biochemically at the age of two years. Since no genetic diagnosis was established then, this was now done as part of a scientific evaluation of the case. The sequencing of the *ALDH5A1* gene revealed compound heterozygosity for a known pathogenic variant as well as a novel variant. Both variants are situated within the highly conserved NAD^+^ binding domain and independently cause a complete loss of enzyme activity. The novel heterozygous missense variant c.728T > C (p.Leu243Pro) results in a substitution of leucine to proline at residue 243. Both leucine and proline belong to the group of non-polar amino acids. Proline, however, is known to have a substantial effect on secondary structures such as α-helices and β-sheets and is often found in protein turns. Due to the presence of a heterocyclic pyrrolidine, proline’s side chain is tightly connected to both the α-carboxyl group and the α-amino group of the amino acid. Thus, the amino group cannot function as a hydrogen acceptor, leading to an immense structural rigidity, thereby disrupting the protein’s secondary structure. While mRNA expression in patient-derived dermal fibroblasts seems to be preserved and comparable to non-SSADHD fibroblasts, a significant reduction in protein expression was observed in Western blot analyses. Interestingly, differences in SSADH protein expression in fibroblasts of two independent healthy control subjects could also be detected in the Western blot. The low amount of SSADH protein detected in the control 2 seems to be sufficient to maintain enzyme function, as no clinical signs of SSADH deficiency were reported in this individual. To our knowledge, there are so far no studies that correlate protein expression in Western blotting with SSADH enzyme activity measurements, but in general, strong variability in residual enzyme activity is reported in congenital metabolic disorders, which rarely correlate with the clinical phenotype and the severity of the disease. Pop et al. were able to show that for 27 of 34 allele variants of the *ALDH5A1* gene, no enzyme activity was detectable in a transient expression system. For the remaining seven variants, residual enzyme activities from 25 to > 100% were described. In stable expression experiments, five of these variants still revealed relatively high SSADH enzyme activity [16]. To what extent the presence of homozygous or compound heterozygous variants of the *ALDH5A1* gene has a direct impact on the SSADH protein expression and especially on the enzyme activity remains the subject of further research and offers potential for innovative therapeutic options such as enzyme replacement therapy, gene therapy, pharmacological chaperones or read-through approaches, which might be able to increase the enzyme activity [6].

Overexpression experiments demonstrated that the p.Leu243Pro variant leads to a profound decrease in the enzyme activity. This is interesting, as the variant has no direct contact with the Rossman fold and thus no direct effect on NAD^+^ binding. It can be postulated that the p.Leu243Pro variant has a significant effect on either the structure or the tetramer formation of SSADH, as no protein expression is found in either the patient or when the variant is overexpressed in a knock out cell line. In silico analyses revealed deleterious effects of the p.Leu243Pro residue replacement, as the calculation of several established pathogenicity scores showed exclusively high values and high probability of destabilizing or deleterious effects on the protein (SIFT score of 0.0 and REVEL score of 0.931). Additionally, the application of the FoldX force field algorithm to calculate energy changes in the wildtype and variant protein revealed a highly destabilizing effect of the novel variant itself, with an equally strong effect when in combination with the known p.Cys93Phe variant.

In silico analysis of all reported *ALDH5A1* missense variants showed a clustering of pathogenic missense variants in the NAD^+^ binding domain, affecting the oligomerization domain, part of which is located at residues 175–176. It can be assumed that only a small fraction of the missense variants lying within the NAD^+^ binding domain have a direct influence on NAD^+^ binding. Rather, it must be considered that, due to its size, the NAD^+^ binding domain contributes to the structural integrity and the stability of the SSADH protein as well as to the tetramer formation. We found a high concordance of different pathogenicity scores that consider structural and conservational aspects. Protein regions with a high density of pathogenic variants correlate strongly with high values of the calculated pathogenicity scores. It becomes evident that both the central and the *C*-terminal part of the SSADH protein contribute to the functionality of the enzyme to a high degree. Pathogenic variants cluster around the center of the NAD^+^ binding domain, whereas variants within the catalytic domain are distributed homogeneously.

Our findings expand the knowledge of structural and functional aspects of the SSADH protein, a pivotal enzyme in the degradation of the neurotransmitter GABA. The in vitro and in silico analyses presented here underline the importance of combined genetic and functional studies to understand the pathomechanisms of congenital metabolic diseases. From our data, a distinct susceptibility of the NAD^+^ binding domain towards pathogenic variants of the *ALDH5A1* gene can be postulated. It should be noted that variants in other areas of the protein (e.g., of the mitochondrial targeting sequence) may be incompatible with cell survival.

In summary, we here present an analysis of the mutational spectrum of the *ALDH5A1* gene and the functional characterization of a previously unknown variant that is causative for SSADHD. By simulating all possible missense variants, we could show that the central and the *C*-terminal regions of the SSADH protein are particularly susceptible to pathogenic variants, while the *N*-terminal region, where the mitochondrial targeting sequence is located, is largely mutation-free in the population of SSADH deficient patients. Whether this is due to the high functional relevance of this protein segment remains to be investigated. Studies using targeted mutagenesis approaches for amino acid changes in the protein domains, for which no patients with pathogenic variants have been found so far, could help to increase our knowledge about the functional relevance of individual residues within the SSADH protein.

## 4. Materials and Methods

### 4.1. Genetic Analysis

For genetic analysis, written informed consent was obtained from the patient and both parents. Genomic DNA was isolated from a patient blood sample using standard procedures. Sanger sequencing was performed for the whole coding sequence including overlapping intronic splice-sites. The variant description is according to the Human Genome Variation Societies’ guidelines and corresponds to transcript ID NM_001080.3; protein ID NP_001071.1.

### 4.2. Ethics Committee Statement and Informed Consent

This study was approved by the ethics committee of the University of Heidelberg (PaNeM, # S-523/2015) and registered at the German Clinical Trials Register (# DRKS00010150). For all genetic analysis, written informed consent was obtained from the patient and parents or patient’s legal representatives.

### 4.3. PCR Analysis

Total RNA extraction was conducted using Trizol^®^ (Invitrogen, Karlsruhe, Germany) according to the manufacturer’s protocol. An amount of 1 µg RNA was mixed with DNase I (Invitrogen) to ensure DNA removal before reverse transcription using 0.5 µg RNA with SuperScript^®^ III Reverse Transcriptase according to the manufacturer’s protocol. Due to the high GC content of the *ALDH5A1* transcript, GC Enhancer and GC Buffer (New England Biolabs, Frankfurt am Main, Germany; #B9026A and #B9023S) were used for *ALDH5A1* PCR reactions. For all PCR reactions, One*Taq*^®^ DNA Polymerase was used with the following primer combinations: *ALDH5A1* (forward: AGCTGATGTTGGGTTAGCAGGT, reverse: CGGACTGCTTCACTCCACCA) and *GAPDH* (forward: GCAAATTCCATGGCACCG, reverse: GAGGCAGGGATGATGTTC).

### 4.4. Semi-Quantitative Protein Analysis

Patient and control fibroblasts were cultured in Dulbecco’s modified Eagle’s medium supplemented with 10% fetal bovine serum, 1% non-essential amino acids, and 1% penicillin/streptomycin at 37 °C and 5% CO_2_. For semi-quantitative analysis of protein concentrations, the cells were collected, washed in phosphate-buffered saline, and resolved in RIPA Lysis and Extraction Buffer (Thermo Fischer Scientific, Darmstadt, Germany Cat. no. 89900). An amount of 30 µg of protein were separated on a 10% polyacrylamide gel and transferred via semi-dry blotting onto a nitrocellulose membrane (GE Healthcare, Braunschweig, Germany). The membrane was blocked for 1 h at room temperature in 5% milk powder in tris-buffered saline and tween (TBS-T, 0.1%). Primary antibodies against SSADH (Abcam, ab129017; rabbit anti-human; dilution 1:5000 in TBS-T) and Vinculin (Invitrogen, #700062; rabbit anti-human; dilution 1:1000 in TBS-T) were incubated at 4 °C overnight. Secondary horseradish peroxidase-conjugated antibody (Dianova, Hamburg, Germany; goat anti-rabbit; dilution 1:10,000 in TBS-T) was incubated for 1 h at room temperature and enabled detection via a chemiluminescent reaction with Enhanced Chemiluminescence (ECL) Plus Western Blotting reagent (Pierce Biotechnology, Waltham, MA, USA). For the Western blot analysis of overexpression constructs, 2 μg of total protein were separated on a 10% SDS PAGE under reducing conditions, followed by electrotransfer to a nitrocellulose membrane (GE Healthcare, Munich, Germany). After blocking the membrane with 5% non-fat milk (Sigma-Aldrich, St. Louis, MO, USA) in TBS-T (5 mM Tris-Cl, 150 mM NaCl, 0.1% Tween 20, pH 7.5), the membrane was probed with rabbit anti-*ALDH5A1* antibody (1:1000). Afterward, the membrane was incubated with a horseradish peroxidase-labeled secondary antibody (1:5000) Cat. Nr.: P0447 (mouse) and P0448 (rabbit) (Agilent technologies, Santa Clara, CA, USA). The final detection of proteins was performed using an ECL Plus Kit (Thermo Scientific, Waltham, MA, USA). As a control for equal loading of the lanes, the blots were stripped and reprobed using a mouse anti-glyceraldehyde 3-phosphate dehydrogenase (GAPDH) antibody (1:10,000), (Abcam, Cat.Nr.: ab8245).

### 4.5. Generation of the SSADH-Deficient HEK-293T Cell Line

The CRISPR-Cas9 gene editing method described by Ran and colleagues was used to generate the SSADH-deficient HEK293T (SSADH-KO-HEK-293T) cell line [34]. Two oligonucleotides targeting the third exon of the human *ALDH5A1* gene (NM_001080.3) were aligned as a duplex and cloned in the vector PX459 (Addgene: Cat.Nr.: 48139, Watertown, MA, USA). The gRNAs with the sequences 5′-CACCGGATGACTGCAGCCACGCCTA-3′ (fwd) and 5′-AAACTAGGCGTGGCTGCAGTCATCC-3′ (rev) were designed using the E-Crisp design tool (http://www.e-crisp.org). The resulting vector was used for the transfection of HEK293T cells. Single cell clones were produced and screened by Western blotting with an anti-SSADH antibody.

### 4.6. Transient Expression of the Constructs

The wild-type *ALDH5A1* cDNA (1605 bp) was cloned into a pcDNA3.1 mammalian expression vector (Invitrogen, Carlsbad, CA, USA) and used as a template to perform PCR-based site-directed mutagenesis with specific oligonucleotides 5′-CTGGCCCTGGCTGAGCCTGCAAGCCAGGCTGGG-3′ (fwd) and 5′-CCCAGCCTGGCTTGCAGGCTCAGCCAGGGCCAG-3′ (rev) to create a p.Leu243Pro mutated cDNA. The nucleotide changes in the clones after transformation were confirmed by Sanger sequencing. SSADH-KO-HEK-293T cells were seeded onto 6-well tissue culture plates in Dulbecco’s Modified Eagle’s Medium (Invitrogen Life Technologies) supplemented with 10% fetal bovine serum (Thermo Fisher Scientific, Waltham, MA, USA) and 1% Penicillin/Streptomycin. After reaching 60–70% confluence, the cells were transfected with MACSfectin reagent (Miltenyi Biotec, Bergisch Gladbach, Germany) according to the manufacturer’s protocol. After 48 h, the cells were solubilized in lysis buffer (50 mM Tris pH 7.4; 150 mM NaCl; 2 mM ethylenediamine-tetraacetic acid (EDTA); 1% NP-40) and the protein concentration was determined by Bradford assay (Biorad, Hercules, CA, USA) according to the manufacturer’s instructions.

### 4.7. SSADH Enzyme Activity

The enzymatic activity of the wildtype and the mutant variant was evaluated using the fluorometric assay described by Gibson and colleagues, [7] with minor adjustments. The cells were first rinsed 3× with *phosphate*-buffered saline (phosphate-buffered saline (PBS), 137 mM NaCl, 2.7 mM KCl, 10 mM Na_2_HPO_4_, 2 mM KH_2_PO_4_) and solubilized in lysis buffer (50 mM Tris pH 7.4; 150 mM NaCl; 2 mM EDTA; 1% NP-40). An amount of 10 μL from the cell lysate was used for the enzyme activity assay, and each sample was processed in duplicate. The incubations were performed for 40 min at room temperature, in 90 μL reaction volume containing: 90 mmol/L Tris pH 8.4, 0.2 mmol/L SSA, 3 mmol/L NAD^+^. Fluorescence was measured at an excitation wavelength of 355 nm and an emission wavelength of 470 nm with the TECAN Infinite Microplate Reader (Tecan Group, Männedorf, Switzerland).

### 4.8. Statistics

The statistical analysis was performed using GraphPad Prism software (v. 5.02, La Jolla, CA, USA). The data are presented as arithmetic means ± SD unless otherwise stated. Differences between two groups were tested using a Student’s *t*-test. A *p-*value < 0.05 was considered statistically significant.

### 4.9. In Silico Analysis of Missense Variants in ALDH5A1

All possible missense variants of the *ALDH5A1* gene were computed and translated to the coding sequence of the *ALDH5A1* genomic region of the GRCh37/hg19 reference genome. The variant call format file was then submitted to the variant effect predictor tool of Ensembl Release 75 (http://grch37.ensembl.org/Tools/VEP; version numbers: CADD v. 1.4, M-CAP v. 3.5a, PolyPhen-2 v. 2.2.2, SIFT v. 5.2.2, and REVEL v. 3.5a). We derived common Ensembl computational scores (CADD, M-CAP, PolyPhen, SIFT, and REVEL) to predict the pathogenic effect of missense variants. The resulting database was fitted to the NM_001080.3 transcript and loaded into RStudio (version 1.2.1335, RStudio, Inc.) with the ggplot2 library loaded. We drew each score along the secondary structure of the SSADH protein to identify domains with clusters of highly pathogenic variants using the geom_smooth function. The database can be obtained upon request from the authors.

### 4.10. FoldX Calculation of Mutamer Stability and Free Energy Change

FoldX suite [35,36] (Version 4.0) was used to calculate the free energy change between wildtype and variant protein (ΔΔ*G*_change_ *=* Δ*G*_mut_ − Δ*G*_WT_). The *RepairPDB* tool of the FoldX suite was performed on the crystal structure of reduced SSADH (PDB accession code: 2w8o) using the Yasara graphic user interface in order to optimize the 3D model’s potential problems through rearrangement of the side chains (e.g., minimizing potential steric clashes) [35]. Afterwards, the *BuildModel* function was used to introduce the desired amino acid substitution in the SSADH structure and to predict the resulting (stabilizing or destabilizing) free energy variations (ΔΔ*G*_change_). Ten independent runs were performed for each potential different tetramer resulting from the random interaction of variant monomers. A positive ΔΔ*G*_change_ implies a destabilizing substitution while a negative value implies a stabilizing substitution with an error margin of approximately 0.46 kcal/mol.

## Figures and Tables

**Table 1:** FoldX analysis of amino acid changes on tetramer stability.

Configuration	Combination	∆∆*G* (kcal/mol)	St.Dev. ∆∆*G*
Monomers	F	7.20	±1.69
	P	7.63	±0.62
Tetramers	FFFF	28.09	±3.32
	PPPP	29.36	±1.11
	FPPP	29.09	±1.94
	FFPP	27.32	±3.21
	FFFP	26.41	±3.36
	FPFP	26.74	±2.16
	PFFP	28.16	±3.32

F = p.Cys93Phe, P = p.Leu243Pro.

**Table 2:** Reported *ALDH5A1* missense variants and corresponding pathogenicity scores.

No.	Genetic Variant	Protein Change	Domain	SIFT	Poly-Phen	REVEL	Source
1	c.106G > C	p.Gly36Arg	Mitochondrial targeting signal	0.82	0	0.103	LOVD [14,15]
2	c.275A > G	p.Asp92Gly	NAD^+^ binding domain	0.03	0.964	0.477	[16]
3	c.277T > C	p.Cys93Arg	NAD^+^ binding domain	0.05	0.979	0.677	[16]
4	c.278G > T	p.Cys93Phe	NAD^+^ binding domain	0.16	0.972	0.638	[12]
5	c.354G > C	p.Lys118Arn	NAD^+^ binding domain	0.01	0.514	0.550	[16]
6	c.371T > G	p.Leu124Arg	NAD^+^ binding domain	0	0.986	0.938	[16]
7	c.412C > T	p.Leu138Pro	NAD^+^ binding domain	0	1	0.948	[17]
8	c.416C > A	p.Ala139Asp	NAD^+^ binding domain	0	0.999	0.886	[18]
9	c.431C > A	p.Ala144Asp	NAD^+^ binding domain	0.08	0.854	0.522	[16]
10	c.466G > A	p.Glu156Lys	NAD^+^ binding domain	0	1	0.924	[19]
11	c.496T > C	p.Trp166Arg	NAD^+^ binding domain	0	0.998	0.970	[20]
12	c.517C > T	p.Arg173Cys	NAD^+^ binding domain	0	1	0.836	[19]
13	c.526G > A	p.Gly176Arg	Oligomerization domain	0	1	0.953	[12]
14	c.527G > A	p.Gly176Glu	Oligomerization domain	0	1	0.924	[21]
15	c.536T > A	p.Ile179Asn	Oligomerization domain	0	0.984	0.765	[16]
16	c.538C > T	p.His180Tyr	Oligomerization domain	0.1	0	0.125	[22]
17	c.545C > T	p.Pro182Leu	Oligomerization domain	0.05	0.735	0.304	[22]
18	c.559C > G	p.Arg187Gly	Oligomerization domain	0	0.942	0.552	[16]
19	c.581C > T	p.Pro194Leu	Oligomerization domain	0	1	0.938	[16]
20	c.587G > A	p.Gly196Asp	Oligomerization domain	0	1	0.958	[23]
21	c.589G > A	p.Val197Met	NAD^+^ binding domain	0	1	0.940	[20]
22	c.608C > G	p.Pro203Arg	NAD^+^ binding domain	0	1	0.941	[16]
23	c.608C > T	p.Pro203Leu	NAD^+^ binding domain	0	1	0.936	LOVD [14,15]
24	c.620C > T	p.Pro207Leu	NAD^+^ binding domain	0	0.998	0.952	[16]
25	c.622A > C	p.Ser208Arg	NAD^+^ binding domain	0.2	0.993	0.686	[16]
26	c.637C > G	p.Arg213Gly	NAD^+^ binding domain	0	0.985	0.699	[16]
27	c.638G > T	p.Arg213Leu	NAD^+^ binding domain	0	0.308	0.859	[24]
28	c.653C > A	p.Ala218Asp	NAD^+^ binding domain	0	0.952	0.941	[16]
29	c.667T > C	p.Cys223Arg	NAD^+^ binding domain	0	0.936	0.867	[25]
30	c.668G > A	p.Cys223Tyr	NAD^+^ binding domain	0	0.983	0.806	[12]
31	c.685C > T	p.Pro229Ser	NAD^+^ binding domain	0	0.961	0.766	[16]
32	c.691G > A	p.Glu231Lys	NAD^+^ binding domain	0.02	0.092	0.693	[21]
33	c.698C > T	p.Thr233Met	NAD^+^ binding domain	0	0.999	0.845	[12]
34	c.709G > A	p.Ala237Thr	NAD^+^ binding domain	0.01	0.293	0.798	[16]
35	c.709G > T	p.Ala237Ser	NAD^+^ binding domain	0	0.492	0.691	[22]
36	c.728T > C	p.Leu243Pro	NAD^+^ binding domain	0	0.999	0.931	This report
37	c.754G > T	p.Gly252Cys	NAD^+^ binding domain	0	1	0.845	[16]
38	c.755G > T	p.Gly252Val	NAD^+^ binding domain	0	1	0.825	[16]
39	c.764A > G	p.Asn255Ser	NAD^+^ binding domain	0.21	0.998	0.673	[12]
40	c.800T > G	p.Val267Gly	NAD^+^ binding domain	0.02	0.998	0.893	[26]
41	c.803G > A	p.Gly268Glu	NAD^+^ binding domain	0	1	0.865	[12]
42	c.851G > A	p.Gly284Asp	NAD^+^ binding domain	0	1	0.989	[16]
43	c.901A > G	p.Lys301Glu	NAD^+^ binding domain	0	1	0.931	[27]
44	c.961G > A	p.Val321Met	Catalytic domain	0.01	0.602	0.421	Faruq et al. 2020, submitted
45	c.1005C > A	p.Asn335Lys	Catalytic domain	0	0.999	0.856	[12]
46	c.1106G > A	p.Arg369His	Catalytic domain	0.16	0.003	0.304	LOVD [14,15]
47	c.1145C > T	p.Pro382Leu	Catalytic domain	0	1	0.805	[12]
48	c.1145C > A	p.Pro382Gln	Catalytic domain	0	1	0.819	[12]
49	c.1226G > A	p.Gly409Asp	Catalytic domain	0	1	0.936	[12]
50	c.1267A > T	p.Thr423Ser	Catalytic domain	0.01	0.998	0.908	[28]
51	c.1274T > C	p.Leu425Pro	Catalytic domain	0	1	0.928	[26]
52	c.1294A > C	p.Met432Leu	Catalytic domain	0	0.827	0.877	[29]
53	c.1321G > A	p.Gly441Arg	Catalytic domain	0	1	0.860	[18]
54	c.1324C > T	p.Pro442Ser	Catalytic domain	0	1	0.961	[16]
55	c.1460T > A	p.Val487Glu	Catalytic domain	0.03	0.989	0.776	[30]
56	c.1478A > G	p.Asn493Ser	Catalytic domain	0	0.997	0.871	[16]
57	c.1498G > C	p.Val500Leu	Catalytic domain	0.08	0.113	0.456	[31]
58	c.1508C > G	p.Pro503Arg	Catalytic domain	0	0.933	0.829	[16]
59	c.1529C > T	p.Ser510Phe	NAD^+^ binding domain	0	1	0.908	[32]
60	c.1547G > A	p.Gly516Glu	NAD^+^ binding domain	0	1	0.951	[33]
61	c.1592G > A	p.Cys531Tyr	Oligomerization domain	0	0.971	0.869	[16]
62	c.1597G > A	p.Gly533Arg	Oligomerization domain	0	1	0.890	[12]

## Abbreviations

CSF	Cerebrospinal fluid
ECL	Enhanced chemiluminescence
GABA	γ-amino butyric acid
GABAT	GABA transaminase
GAD	Glutamate decarboxylase
GAPDH	Glyceraldehyde 3-phosphate dehydrogenase
GHB	γ-hydroxybutyrate
LOVD	Leiden Open Variation Database
NAD^+^	Nicotinamide adenine dinucleotide
PBS	Phosphate-buffered saline
PCR	Polymerase chain reaction
PISA	Proteins, Interfaces, Structures and Assemblies
SSA	Succinic semialdehyde
SSADH	Succinic semialdehyde dehydrogenase
SSADHD	Succinic semialdehyde dehydrogenase deficiency
SSR	Succinic semialdehyde reductase
SUDEP	Sudden unexpected death in epilepsy
TBS-T	Tris-buffered saline and tween
TCA	Tricarboxylic acid
VEP	Variant Effect Prediction
WT	Wild-type
